# Distinctive Features of Phubbing Behavior and Being Phubbed in Adolescents: Empathy, Social Cognition, Interpersonal Competence, and Smartphone Addiction

**DOI:** 10.3390/bs16101847

**Published:** 2026-10-09

**Authors:** Esra Sizer, Remzi Oğulcan Çıray, Eren Halaç, Yeliz Balca Kaya, Hozan Saatçioğlu, İbrahim Sinan Buğur

**Affiliations:** 1 Department of Child and Adolescent Psychiatry, Faculty of Medicine, Mardin Artuklu University, 47100 Mardin, Türkiye; hozansaatcioglu@artuklu.edu.tr; 2 Department of Child and Adolescent Psychiatry, Faculty of Medicine, Dokuz Eylül University, 35100 İzmir, Türkiye; ogulcan.ciray@deu.edu.tr; 3 Child and Adolescent Psychiatry, Mardin Training and Research Hospital, 47100 Mardin, Türkiye; halac.eren@gmail.com; 4 Child and Adolescent Psychiatry, Malatya Training and Research Hospital, 44100 Malatya, Türkiye; balcayeliz@gmail.com; 5 Department of Child Health and Diseases, Faculty of Medicine, Atılım University, 06100 Ankara, Türkiye; sinan.bugur@atilim.edu.tr

**Keywords:** phubbing, empathy, social cognition, adolescent

## Abstract

The aim of our study is to concurrently examine, within the same structural model, the effects of social cognitive functions, empathy, emotional regulation difficulties, interpersonal competence and smartphone addiction on adolescents who display phubbing behavior and those who are being phubbed. A cross-sectional study was carried out with the participation of 305 adolescents between the ages of 16 and 18. The Difficulties in Emotion Regulation Scale—Short Form, Interpersonal Reactivity Index, Interpersonal Competence Scale, Generic Scale of Phubbing and Generic Scale of Being Phubbed, Smartphone Addiction Scale and Reading the Mind in the Eyes Test were administered to the participants. Smartphone addiction was positively associated with phubbing behavior (β = 0.775, *p* < 0.001), whereas interpersonal competence was negatively associated with phubbing (β = −0.145, *p* < 0.001). A significant positive indirect association between emotional regulation difficulties and phubbing behavior via smartphone addiction was observed (β = 0.273, *p* < 0.001). Interpersonal reactivity was positively associated with being phubbed (β = 0.203, *p* = 0.001), whereas social cognition was not significantly associated with either outcome. Our findings suggest that different socio-cognitive competencies and smartphone addiction may be associated with phubbing in adolescents, and that these associations may differ between phubbing behavior and being phubbed.

## 1. Introduction

In recent years, with the rapid advance of technology, smartphones have become an indispensable part of daily life, not only by enabling individuals to connect with others but also due to the continuously increasing services they provide in areas such as gaming, entertainment, education, and health. It has been observed that, especially among adolescents, the rate of smartphone usage has reached exceptionally high levels: around 94–95% (Auhuber et al., 2019; Center, 2024). However, studies have indicated that, in addition to these advantages, smartphone use may also lead to negative effects both mentally and physically, as well as disruptions in face-to-face social communication (Basu & Mukherjee, 2021; Davey & Davey, 2014; Ivanova et al., 2020).

In the context of smartphone use disrupting social interactions, the concept of phubbing comes to the fore. Phubbing is a situation in which individuals engaged in face-to-face social interaction neglect the person they are conversing with by focusing on their phones, and the other party perceives this as rude and dismissive behavior, experiencing negative affect as a result (Ugur & Koc, 2015). In terms of the development and continuity of interpersonal relationships, attentive listening, eye contact, and body language in face-to-face communication are of great importance. According to the expectancy violation theory, distraction during dialogue makes it more difficult to perceive social cues, and this leads to deterioration in relationship quality during conversation and a violation of expectations regarding appropriate behaviors (Burgoon & Jones, 1976; Chotpitayasunondh & Douglas, 2018b). Additionally, in a person exposed to phubbing, the sense of being excluded from conversation and being ignored may lead to certain emotional responses and maladaptive coping responses in the short and long term (Vanden Abeele et al., 2024; Williams, 1997). It has also been stated that the individual who exhibits phubbing behavior may be negatively evaluated by the person they are communicating with due to the act of ignoring, and over time this situation may become a cause of social exclusion for both parties (Chotpitayasunondh & Douglas, 2018b; Vanden Abeele et al., 2024).

It is important to also consider social–cognitive and interpersonal processes in understanding phubbing. Social cognition involves the individual’s ability to gather and recognize information from the social environment and to develop appropriate behaviors accordingly; it requires perceiving verbal and non-verbal communication elements (such as facial expressions, body language, and tone of voice) and processing these social signals. The interpretation of these social signals is a holistic mental process that shapes the individual’s motivation, emotional responses, and context-appropriate behaviors (Adolphs, 2001). Empathy and theory of mind are also components of these social cognitive abilities. Empathy refers to the ability to understand other individuals on a deeper emotional level and to connect with them. This encompasses not only the capacity to recognize an individual’s basic needs, but also the ability to perceive and internalize their perspective (Davis, 1983; Deutsch & Madle, 1975). Unlike socio-emotional processes, theory of mind encompasses the process of inferring and assessing what others think, believe, intend, or feel based on available social cues (Frith & Frith, 2005; Kanske et al., 2015). Individual differences in these skills may influence the extent to which a person is sensitive to the social and emotional responses of others during face-to-face communication, as well as how they evaluate the consequences of phone use on interaction. Emotional regulation and interpersonal competence also play a significant role in sustaining social interactions. Interpersonal competence is the sum of social skills that include initiating, adapting, and maintaining communication, providing emotional support, self-disclosure, conflict management, and coping with problem situations (Buhrmester et al., 1988). Similarly, emotional regulation skills help individuals recognize and manage the emotional responses that arise during interpersonal interaction (Gratz & Roemer, 2004). Difficulties in these areas may be associated with reduced engagement in face-to-face interactions and greater reliance on smartphones during social situations. In contrast, higher interpersonal competence may contribute to sensitivity to social cues and the maintenance of reciprocal interaction during communication (Alsawalqa & Al-Zawahreh, 2025; Rahmani et al., 2024).

Smartphone addiction may play an important role in explaining the potential indirect associations between these individual and interpersonal characteristics and phubbing behavior. According to previous studies and the Compensatory Internet Use Theory, individuals may turn to digital technologies in order to cope with negative emotional experiences or to distance themselves from difficulties encountered in interpersonal interactions (Cheng et al., 2024; Kardefelt-Winther, 2014; Park et al., 2021). In addition, previous studies have reported that longer daily smartphone use may be associated with problematic use symptoms such as loss of control, compulsive use, and functional impairment (Park et al., 2020, 2021). Based on this information, it was hypothesized that emotional regulation difficulties, interpersonal competence, and daily smartphone use duration may be associated with smartphone addiction, and that smartphone addiction may play a mediating role in the potential indirect associations between these variables and phubbing behavior. Although previous studies have examined the relationship between smartphone addiction and phubbing, as well as various psychological and interpersonal correlates of phubbing, most have primarily focused on phubbing behavior, whereas comparatively less attention has been given to characteristics associated with being phubbed (Aagaard, 2020; Chotpitayasunondh & Douglas, 2018b; Erzen et al., 2021; Öztekin & Yıldırım, 2025). Phubbing behavior and being phubbed constitute two related but conceptually distinct dimensions of interpersonal interaction (Chotpitayasunondh & Douglas, 2018a). An adolescent may display phubbing behavior during face-to-face communication, while also being exposed to similar behavior from others. However, the experience of being phubbed may be associated not only with the other person’s phone use, but also with the characteristics of the individual reporting this experience. Therefore, evaluating both dimensions together within the same sample may contribute to our understanding of the similarities and differences in the psychosocial characteristics associated with phubbing behavior and being phubbed. Given this gap in the literature, it was decided to evaluate emotional regulation difficulties, interpersonal competence, daily smartphone use duration, smartphone addiction, interpersonal reactivity, and social cognition together within the same structural model. Using this model, the aim was to simultaneously examine the direct associations of these variables with phubbing behavior and being phubbed, as well as the potential indirect associations of emotional regulation difficulties, interpersonal competence, and daily smartphone use duration through smartphone addiction. To the best of our knowledge, no study has jointly evaluated the direct and indirect associations of these variables with phubbing behavior and being phubbed within the same structural model in adolescents. Accordingly, the aim of our study is to examine the shared and distinct psychosocial association patterns of phubbing behavior and being phubbed among adolescents.

## 2. Materials and Methods

The cross-sectional and correlational study was conducted between 31 August 2025 and 31 December 2025 by including adolescents aged 16–18, regardless of gender, who owned at least one smartphone and presented for the first time to the pediatric outpatient clinics of the Mardin Training and Research Hospital, Mardin, Turkey, for routine medical consultations, and who did not have psychiatric or physical illnesses, using a consecutive sampling method. This setting was selected to recruit non-psychiatric adolescents from a routine clinical population rather than from a psychiatric treatment setting. All eligible adolescents were invited to participate in the study in order of admission and were consecutively included, thus aiming to minimize selection bias. They were required to be within the target age range and possess sufficient literacy to complete the questionnaires. Adolescents were excluded from the study if they had a history of psychiatric diagnosis as identified through medical records or self-report, had left more than 20% of questionnaire items unanswered, exhibited inconsistent response patterns, or did not complete the consent process. During the study period, all 530 adolescents aged 16–18 years were invited to the study in the order of presentation. Of these participants, 113 did not agree to participate in the study, 70 were excluded from the study because they had a current or previous psychiatric history, and 42 participants were excluded because they left more than 20% of the questionnaire items unanswered or showed inconsistent response patterns. Subsequently, the following instruments were administered to the participants as data collection tools: the sociodemographic data form, the Difficulties in Emotion Regulation Scale—Short Form, the Interpersonal Reactivity Index, the Interpersonal Competence Questionnaire, the Generic Scale of Phubbing and the Generic Scale of Being Phubbed Scale, the Smartphone Addiction Scale—Short Form, and the Reading the Mind in the Eyes Test.

Difficulties in Emotion Regulation Scale—Short Form (DERS): A 16-item self-report scale that assesses difficulties in recognizing, accepting, and managing emotions. The original Difficulties in Emotion Regulation Scale developed by Gratz and Roemer (2004) formed the basis for the short form created by Bjureberg et al. (2016). It consists of the subdimensions clarity, goals, impulse, strategy, and acceptance. High scores indicate increased difficulty in emotional regulation. In the validity and reliability study of the Turkish version, the total internal consistency coefficient of the scale was calculated as 0.92 (Sarıtaş-Atalar et al., 2015). In the present sample, Cronbach’s alpha for the DERS-16 was 0.95.

Interpersonal Reactivity Index (IRI): A multidimensional self-report scale that assesses individuals’ empathic tendencies. It consists of four subdimensions—empathic concern, perspective taking, fantasy, and personal distress—and includes a total of 28 items (Davis, 1983). The adaptation of the scale into Turkish, its validation, and psychometric evaluation studies were conducted by Engeler and Yargıç (2007). In the present sample, Cronbach’s alpha for the IRI was 0.73.

Interpersonal Competence Scale (ICS): A scale developed by Buhrmester et al. to evaluate individuals’ interpersonal skills in their social relationships, consisting of a total of 40 items. The scale comprises five subdimensions, each with eight items: initiating relationships, emotional support, assertiveness, self-disclosure, and conflict management (Buhrmester et al., 1988). The internal consistency coefficient of the scale in the Turkish adaptation, validity, and reliability study was found to be 0.94 for the scale (Baytemir, 2016). In the present sample, Cronbach’s alpha for the Interpersonal Competence Scale was 0.90.

Generic Scale of Phubbing (GSP) and the Generic Scale of Being Phubbed (GSBP) Scale: Self-report instruments developed to assess the effects of mobile phone use in social settings on interpersonal relationships. These scales aim to measure individuals’ tendencies to use their phones by ignoring others (phubbing behavior) and their experiences of being exposed to such behavior by others (being phubbed) (Chotpitayasunondh & Douglas, 2018a). The Cronbach’s alpha internal consistency coefficients of the scales adapted into Turkish and evaluated for their psychometric properties were found to be 0.78 and 0.87, respectively (Göksün, 2019). In the present sample, Cronbach’s alpha was 0.92 for the GSP and 0.93 for the GSBP.

Smartphone Addiction Scale Short Form: A 10-item, 6-point Likert-type (1: strongly disagree–6: strongly agree) self-report scale developed by Kwon et al. to assess individuals’ levels of addiction to smartphone use. The scale encompasses various dimensions, including disturbance of daily life activities, withdrawal, tolerance, virtual relationships, overuse, and positive expectations (Kwon et al., 2013). Within the scope of the validity and reliability studies of the Turkish form, the Cronbach’s alpha internal consistency coefficient was found to be 0.87 (Noyan et al., 2015).

Reading the Mind in the Eyes Test (RMET): A visual-based performance test developed by Baron-Cohen and colleagues to measure theory of mind skills. The test consists of 36 items. In each item, a black-and-white image showing only the eye region is presented, and the participant is asked to choose the word that best describes what the person is thinking or feeling from among four options. Each correct answer is scored as one point, the total score ranges from 0 to 36, and higher scores indicate a better ability to infer mental states from the eyes (Baron-Cohen et al., 2001). The Turkish validity and reliability study of the scale was conducted by Girli, and the Cronbach’s alpha was found to be 0.71 (Girli, 2014).

### Statistical Analysis

Statistical analyses were performed using SPSS version 31.0 (SPSS Inc., Chicago, IL, USA) and RStudio (lavaan package 0.6-21) for structural equation modeling. Whether the data were normally distributed or not was evaluated by visual inspection of skewness–kurtosis coefficients, histograms, and Q–Q plot graphs. Continuous variables were presented as mean, standard deviation, and minimum–maximum values; categorical variables were presented as counts and percentages. For comparisons of continuous variables, the independent sample *t*-test was used; for comparisons of categorical variables, the Pearson chi-square test was used. The effect size of differences between groups for continuous variables was indicated by the Cohen’s d coefficient. Relationships between scale scores were examined using Pearson correlation analysis. Potential multicollinearity among the predictors was assessed using tolerance and variance inflation factor (VIF) statistics. For the final structural path model, the sample size was additionally evaluated by considering model complexity and methodological recommendations in the SEM literature (Kline, 2016; Wolf et al., 2013). The final sample of 305 adolescents was considered reasonable for estimating the observed-variable structural path model.

In the structural path model, the direct and indirect relationships among emotional regulation difficulties, interpersonal competence, daily phone usage duration, smartphone addiction, interpersonal reactivity, social cognition, phubbing behavior, and being phubbed were examined. Smartphone addiction was specified as a mediator of the associations of emotional regulation difficulties, interpersonal competence, and daily phone usage duration with phubbing behavior and being phubbed. Direct paths from smartphone addiction, emotional regulation difficulties, interpersonal competence, daily phone usage duration, interpersonal reactivity, and RMET scores to both phubbing outcomes were estimated within the same model. Because phubbing behavior and being phubbed represent related interpersonal experiences, a residual covariance was specified between the two outcome variables to account for shared unexplained variance.

Missingness in the final analytic sample was minimal; across the scale-based variables included in the structural model, only two isolated missing observations (0.65%, corresponding to different single participants across scales) were present per variable. These remaining missing observations were handled using full-information maximum likelihood (FIML) under the missing-at-random assumption. Model parameters were estimated using maximum likelihood. Standard errors and 95% confidence intervals for direct, indirect, and total effects were calculated using the bootstrap method with 5000 resamples. In assessing the theoretical model, a full-mediation model was initially tested. Subsequently, a partial-mediation model was constructed by adding direct paths from emotional regulation difficulties, interpersonal competence, and daily smartphone use to both phubbing behavior and being phubbed. The comparison of the two nested models was based on the chi-square difference test, conventional fit indices (CFI, TLI, RMSEA, and SRMR), and information criteria (AIC and BIC). Because these indices may favor different aspects of model performance and parsimony, the selection of the final model was not based on a single fit statistic. Rather, the partial-mediation model was retained because it allowed the simultaneous estimation of both direct and indirect associations within the proposed cross-sectional path framework. The primary research questions and the examination of indirect associations were specified during the initial study planning. The final integrated path model was subsequently developed during the analysis stage to estimate direct and indirect associations with both phubbing outcomes simultaneously. Age, sex, and socioeconomic status were evaluated as potential covariates. Their inclusion did not materially alter the direction, magnitude, or statistical significance of the principal path estimates, and none was significantly associated with the endogenous outcomes. Therefore, the more parsimonious model without these covariates was retained. In all analyses, two-tailed tests were conducted and *p* < 0.05 was considered statistically significant.

## 3. Results

### 3.1. Sample Characteristics

A total of 305 adolescents were included in the study. Of the participants, 50.8% were female (*n* = 155) and 49.2% were male (*n* = 150). The mean age for male participants was 16.38 ± 0.68 years, and for female participants it was 16.42 ± 0.68 years. No significant difference in age was found between genders (*p* = 0.615). The demographic information of the participants is provided in Table 1. No significant differences were found between genders in terms of educational level, maternal age, paternal age, or daily phone usage duration (respectively, *p* = 0.205, *p* = 0.624, *p* = 0.275, *p* = 0.734). The participants’ average daily smartphone usage duration was 5.41 ± 3.71 h. Based on the cut-off score of the Smartphone Addiction Scale–Short Form, 109 participants (35.7%) scored above the screening threshold, including 55 males (36.6%) and 54 females (34.8%). No statistically significant difference was observed between genders in terms of smartphone addiction (*p* = 0.739). When scale scores were evaluated by gender, it was observed that the DERS scale score was significantly higher in girls compared to boys (*p* = 0.009). The comparison of scale scores is presented in detail in Table 1.

### 3.2. Correlation Analysis

It was found that the level of phubbing behavior was positively correlated with smartphone addiction (r = 0.818, *p* < 0.001) and DERS (r = 0.331, *p* < 0.001) scale scores, and negatively correlated with ICS score (r = −0.325, *p* < 0.001). GSBP level was found to be positively correlated with IRI (r = 0.228, *p* < 0.001), DERS (r = 0.170, *p* = 0.003), smartphone addiction (r = 0.157, *p* = 0.006) and GSP (r = 0.180, *p* = 0.002) scores (Table 2).

### 3.3. Mediation Model

The comparison of the full- and partial-mediation models indicated that the partial-mediation model provided a significantly better fit according to the chi-square difference test (Δχ^2^ = 24.22, Δdf = 6, *p* < 0.001). The partial-mediation model also showed better fit on CFI, TLI, RMSEA, SRMR, and AIC, whereas BIC favored the more parsimonious full-mediation model. Because the partial-mediation model had only two degrees of freedom and was therefore close to saturated, its global fit indices were interpreted cautiously. Accordingly, the selection of the final model was not based on a single fit statistic. The partial-mediation model was retained as a less constrained representation that allowed direct and indirect associations to be estimated simultaneously. No evidence of problematic multicollinearity was observed among the predictors, with VIF values ranging from 1.004 to 1.534 and tolerance values ranging from 0.652 to 0.996.

**Figure 1:**
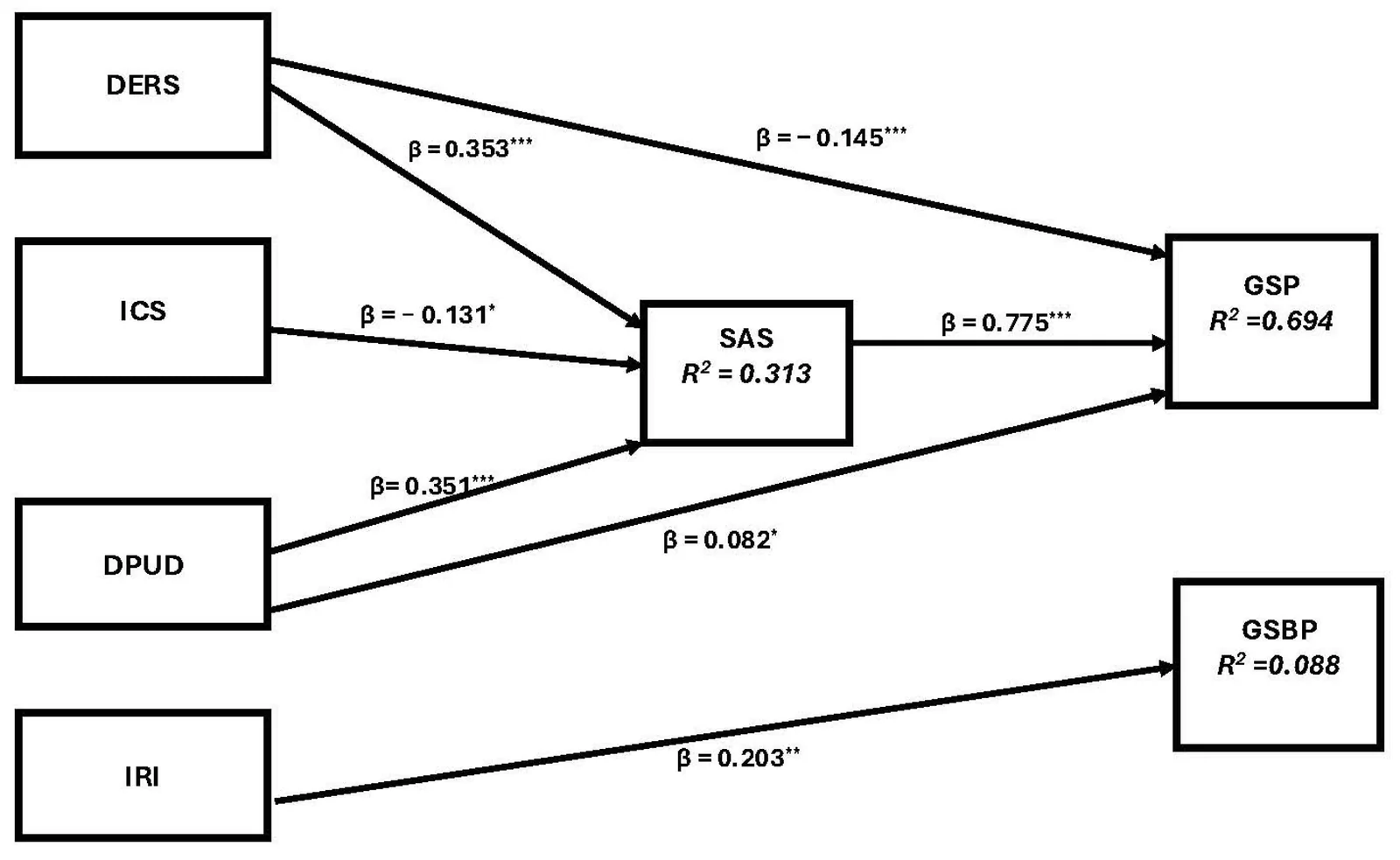
Final structural path model of phubbing behavior. Note. Standardized path coefficients (β) are shown. Only statistically significant direct paths are displayed. * *p* < 0.05, ** *p* < 0.01, *** *p* < 0.001. DERS: Difficulties in Emotion Regulation Scale—Short Form, ICS: Interpersonal Competence Scale, IRI: Interpersonal Reactivity Index, GSBP: Generic Scale of Being Phubbed, GSP: Generic Scale of Phubbing, DPUD: Daily Phone Usage Durationi, SAS: Smartphone Addiction Scale Short Form.

In the final structural path model, it was observed that DERS scores were significantly and positively associated with smartphone addiction scores (B = 0.275, SE = 0.038, β = 0.353, *p* < 0.001, 95% bootstrap CI [0.201, 0.350]). ICS scores were significantly and negatively associated with smartphone addiction (B = −2.975, SE = 1.227, β = −0.131, *p* = 0.015, 95% bootstrap CI [−5.434, −0.617]), whereas daily phone usage duration was significantly and positively associated with smartphone addiction (B = 1.232, SE = 0.198, β = 0.351, *p* < 0.001, 95% bootstrap CI [0.859, 1.639]). It was seen that RMET scores were not significantly associated with either GSP or GSBP (Table 3, Figure 1).

ICS was significantly and negatively associated with GSP (B = −5.270, SE = 1.488, β = −0.145, *p* < 0.001, 95% bootstrap CI [−8.095, −2.369]), while daily phone usage time (B = 0.458, SE = 0.185, β = 0.082, *p* = 0.013, 95% bootstrap CI [0.100, 0.835]) and smartphone addiction (B = 1.237, SE = 0.063, β = 0.775, *p* < 0.001, 95% bootstrap CI [1.113, 1.366]) were significantly and positively associated with GSP. IRI scores were the only variable significantly and positively associated with GSBP (B = 0.444, SE = 0.129, β = 0.203, *p* = 0.001, 95% bootstrap CI [0.185, 0.690]) (Table 4, Figure 1).

### 3.4. Indirect Effects

A significant positive indirect association between DERS and GSP through smartphone addiction was observed (B = 0.341, β = 0.273, SE = 0.053, 95% bootstrap CI [0.239, 0.449], *p* < 0.001). A significant negative indirect association between ICS and GSP through smartphone addiction was also observed (B = −3.680, β = −0.101, SE = 1.553, 95% bootstrap CI [−6.868, −0.752], *p* = 0.018). The daily phone usage duration was observed to have a significant positive indirect association with GSP through smartphone addiction (B = 1.523, β = 0.272, SE = 0.244, 95% bootstrap CI [1.063, 2.017], *p* < 0.001). Detailed data on the indirect effects are presented in Table 5.

### 3.5. Total Effects

It was found that the total association between DERS score and GSP was significantly positive (B = 0.258, β = 0.207, SE = 0.066, 95% bootstrap CI [0.124, 0.387], *p* < 0.001). A significant negative total association between ICS and GSP was also observed (B = −8.950, β = −0.246, SE = 1.984, 95% bootstrap CI [−12.886, −5.039], *p* < 0.001), whereas daily phone usage duration showed a significant positive total association with GSP (B = 1.981, β = 0.353, SE = 0.318, 95% bootstrap CI [1.378, 2.631], *p* < 0.001). The total association of DERS, ICS, and daily phone usage duration on GSBP were not statistically significant (*p* > 0.05, Table 6).

### 3.6. Exploratory Analysis of Interpersonal Reactivity Subdimensions

Following the finding that the total interpersonal reactivity score was significantly and positively associated with being phubbed in the structural model, an exploratory multiple linear regression analysis was conducted to examine which subdimensions were related to this association. The regression model including all four subdimensions simultaneously was statistically significant [F(4, 300) = 5.807, *p* < 0.001, R^2^ = 0.072]. Empathic concern (β = 0.197, *p* = 0.003) and fantasy (β = 0.156, *p* = 0.011) were independently and positively associated with being phubbed, whereas perspective taking (β = −0.050, *p* = 0.430) and personal distress (β = −0.001, *p* = 0.990) were not significantly associated with this outcome.

## 4. Discussion

In our study, factors associated with phubbing behavior and being phubbed among adolescents were examined within the same structural path analysis model. Our most important finding is that smartphone addiction showed the strongest association with phubbing behavior in adolescents. Daily phone usage time showed a positive direct association with phubbing behavior, while interpersonal competence showed a negative direct association. The indirect associations of emotional regulation difficulties, interpersonal competence, and daily phone usage time with phubbing behavior through smartphone addiction were significant. Regarding being phubbed, interpersonal reactivity was the only variable showing a significant direct association in the structural model. Exploratory regression analysis further showed positive associations of empathic concern and fantasy with being phubbed. In addition, the indirect effects of the other variables on being phubbed via smartphone addiction were not found to be significant.

In our study, higher smartphone addiction scores were strongly associated with higher levels of phubbing behavior. In a similar manner to our study findings, Syifa et al. demonstrated in their study with 260 adolescent participants that smartphone addiction is a determinant of phubbing behavior (Syifa & Lestari, 2026). It was also reported in the study by Tufan et al., with 640 university students, that there are positive relationships among fear of missing out, smartphone addiction, and phubbing (Tufan et al., 2026). The observation of findings similar to those obtained in university students in our adolescent sample suggests that the relationship between smartphone addiction and phubbing may also be seen in different developmental periods. In the study conducted by Lai et al., it was also demonstrated that there is a strong positive relationship between smartphone addiction and phubbing, and that phubbing behavior may be associated with psychosocial difficulties such as depression, relationship dissatisfaction, and loneliness (Lai et al., 2023). When these findings are evaluated together, they suggest that smartphone addiction is an important correlate of phubbing behavior. However, given the cross-sectional design of our study, we cannot definitively determine the direction and causality of this relationship.

In our study, it was observed that daily phone usage time has both a direct and an indirect positive predictive effect on phubbing behavior through smartphone addiction. The indirect association through smartphone addiction accounted for approximately 77% of the total statistical association between daily phone usage time and phubbing behavior, whereas the direct association was relatively small. However, the need to use smartphones for communication or purpose-related needs during face-to-face social interactions may increase the likelihood of individuals directing their attention to their phones during conversation (Capilla Garrido et al., 2021). This may partly explain why daily phone usage time remained significantly associated with phubbing even after smartphone addiction was included in the model. However, due to the cross-sectional design of our study, the temporal direction and causality of this relationship cannot be determined; it is important for future longitudinal studies to examine the direction and temporal sequence of these relationships.

In our study, while the direct association between emotional regulation difficulties and phubbing behavior was not significant in the final model, the indirect association through smartphone addiction was significant. This finding indicates a significant indirect association between emotional regulation difficulties and phubbing behavior via smartphone addiction within the proposed cross-sectional path model. Similarly, in the study conducted by Yin et al., a partial mediating effect was demonstrated in the model associating emotional regulation and self-control with smartphone addiction (Yin et al., 2024). Some studies have shown that, especially in young individuals, smartphone use and internet use have become tools for coping with negative emotions, compensating, and controlling or avoiding impulsive behaviors (Alsawalqa & Al-Zawahreh, 2025; Gámez-Guadix, 2014). Adolescents who experience difficulties in emotional regulation may turn to smartphones to cope with negative emotions; this suggests that there may be an association among emotional regulation difficulties, smartphone addiction, and phubbing. However, these coping processes and the temporal sequence among them were not directly tested in the present study. Considering the cross-sectional design of the study, the findings should be interpreted cautiously without making causal inferences.

In our study, in the final model examining the associations of interpersonal competence with being phubbed and phubbing behavior, interpersonal competence showed a negative association with phubbing behavior, both directly and indirectly through smartphone addiction. In the study conducted by Rahmani et al. with Generation Z participants aged 18–22, it was demonstrated that interpersonal competence, which is necessary for effective communication, is negatively affected by phubbing behavior (Rahmani et al., 2024). In the study conducted by Han et al., it was shown that interpersonal competence is negatively correlated with phubbing and negatively predicts phubbing behavior, whereas smartphone addiction positively predicts phubbing behavior (Han et al., 2022). It was also stated in the study conducted by Lai et al. that interpersonal adaptation skills negatively affect phubbing behavior and may have a protective effect against it (Lai et al., 2023). Our findings suggest that adolescents with higher interpersonal competence may find it easier to initiate and maintain face-to-face communication, and this may be associated with a reduced tendency to turn to smartphones during social interactions. In this respect, having strong skills in social self-expression, relationship maintenance, and conflict resolution may serve as a protective psychosocial asset against phubbing behaviors in adolescents, and it may be important to examine its effects on resilience among adolescents in larger, long-term studies.

In our study, the level of interpersonal reactivity was found to be positively associated with being phubbed, while the final model explained 69.4% of the variance in phubbing behavior but only 8.8% of the variance in being phubbed. This marked difference suggests that phubbing behavior and being phubbed may have different psychosocial patterns of association and that the variables included in the present model explain only a limited proportion of the individual variability in being phubbed. In the structural model, interpersonal reactivity was the only variable that showed a significant and positive direct association with being phubbed. In the exploratory regression analysis conducted to evaluate in greater detail which dimensions of empathy were associated with this relationship, the empathic concern and fantasy subdimensions of the Interpersonal Reactivity Index were found to be significantly and positively associated with being phubbed, whereas the perspective-taking and personal distress subdimensions did not show significant associations. This finding suggests that the association identified for the total interpersonal reactivity score in the structural model may be particularly related to the empathic concern and fantasy dimensions. Previous studies have shown that digital device use may adversely affect empathic processes in face-to-face communication and that higher levels of phubbing behavior may be associated with lower empathy and weaker social relationships (Németh et al., 2026; Öztekin & Yıldırım, 2025; Schmidt-Barad & Chernyak-Hai, 2026; Turkle, 2017). In addition, cognitive and emotional empathy have been reported to be associated with emotional regulation in different ways (Thompson et al., 2022), and it has been suggested that individual differences in empathy levels may be related to patterns of visual attention toward social and emotional stimuli (Ma et al., 2026). Taken together, these findings suggest that individual differences related to empathy may be associated with how interpersonal interactions are experienced and reported. However, the explanation that individuals with higher interpersonal reactivity may notice more clearly, or interpret differently, the shift in the other person’s attention toward their phone was not directly tested in the present study. Because visual attentional bias, attribution of intention, perceived rejection, and the other person’s actual phone use behavior were not measured, this possible mechanism should be considered only as a hypothesis to be tested in future research. In addition, given the low explanatory power of the model for being phubbed, future studies with larger samples should examine other interpersonal and psychological variables such as friendship relationships, attachment characteristics, social anxiety, loneliness, and rejection sensitivity together.

In our study, it was found that the RMET score was not significantly associated with phubbing behavior or being subjected to phubbing. The findings concerning problematic technology use and RMET have been inconsistent and limited in number. In the study by Ünal-Aydın et al., although individuals with social media addiction had lower total RMET scores compared to non-addicted individuals, it was noted that this difference remained borderline and was not considered statistically significant (Ünal-Aydın et al., 2020). In another study, no significant difference was found between those with high and low social media addiction in terms of mind-reading ability (Ertuğrul-Yaşar, 2024). In contrast, Sain and colleagues have stated that studies have reported a negative relationship between smartphone addiction and screen time and RMET, but that the effect sizes of these relationships were low (Sain et al., 2026). Only one study has been conducted on phubbing and RMET: in their experimental study using a phone-based neglect manipulation via video with participants aged 18–24, Huang et al. demonstrated that higher levels of phubbing behavior were associated with poorer RMET scores (Shikui, 2022). In our study, the absence of a significant association between RMET scores and phubbing behavior may suggest that the ability to recognize social cues is not necessarily associated with lower levels of phubbing. Even if individuals are able to recognize the emotional states of others, other factors such as smartphone addiction, emotional regulation difficulties, and lower interpersonal competence may be associated with their tendency toward phubbing behavior during face-to-face communication. Therefore, the recognition of social cues alone may not be sufficient to explain individual differences in phubbing behavior.

## 5. Conclusions

The fact that this is a single-center study limits the generalizability of our findings to other populations and cultural contexts. Additionally, given the cross-sectional design, the observed direct and indirect associations do not establish temporal mediation or causal pathways. Reverse or reciprocal relationships remain possible, and longitudinal studies are needed to examine the direction and temporal sequence of the proposed relationships. Furthermore, the psychiatric evaluation of participants was conducted according to exclusion criteria by reviewing self-report scales and treatment records; a structured clinical assessment was not performed, and the possibility of undiagnosed mental conditions should not be overlooked. In addition, the use of self-report scales may also lead to response bias due to factors such as social desirability and individual differences in insight. The exclusion of adolescents with a current or previous psychiatric history may have reduced variability in emotional regulation, problematic smartphone use, and interpersonal functioning, and may limit the generalizability of the findings to adolescents with psychiatric conditions.

Our findings suggest that phubbing behavior and being phubbed may have different psychosocial correlates, with the present model explaining substantially more variance in phubbing behavior than in being phubbed. Smartphone addiction, emotional regulation difficulties, and interpersonal competence showed significant direct or indirect associations with phubbing behavior, whereas interpersonal reactivity was the only significant direct correlate of being phubbed in the structural model. RMET scores were not significantly associated with either outcome. In a world where smartphone use continues to increase, it is recommended that future studies with larger samples be designed to better understand phubbing behavior and its consequences in order to identify and inform the development of strategies aimed at developing and maintaining face-to-face social communication skills in adolescents.

## Figures and Tables

**Table 1:** Comparison of DERS, ICS, IRI, GSBP, GSP, RMET, and SAS scores by gender.

Variable	Male (*n* = 150) Mean ± SD	Female (*n* = 155) Mean ± SD	*t*	*p*	Cohen’s d
DERS	47.46 ± 17.41	52.42 ± 15.68	−2.61	0.009	−0.30
ICS	3.06 ± 0.60	2.97 ± 0.54	1.25	0.213	0.14
IRI	83.62 ± 12.20	85.91 ± 11.49	−1.68	0.093	−0.19
GSBP	72.81 ± 23.91	73.75 ± 27.81	−0.32	0.752	−0.04
GSP	47.62 ± 19.92	45.96 ± 21.61	0.70	0.487	0.08
RMET	21.61 ± 3.11	21.79 ± 2.70	−0.54	0.589	−0.06
SAS	26.24 ± 12.03	26.57 ± 13.99	−0.22	0.823	−0.03

Note. DERS: Difficulties in Emotion Regulation Scale—Short Form, ICS: Interpersonal Competence Scale, IRI: Interpersonal Reactivity Index, GSBP: Generic Scale of Being Phubbed, GSP: Generic Scale of Phubbing, RMET: Reading the Mind in the Eyes Test, SAS: Smartphone Addiction Scale Short Form. Cohen’s d was reported as the effect size. *p* < 0.05 was considered significant.

**Table 2:** Correlations between psychological variables.

Variable	1	2	3	4	5	6	7
1. GSP	—						
2. GSBP	0.180 **	—					
3. SAS	0.818 ***	0.157 **	—				
4. DERS	0.331 ***	0.170 **	0.421 ***	—			
5. IRI	0.042	0.228 ***	0.032	0.311 ***	—		
6. ICS	−0.325 ***	−0.018	−0.263 ***	−0.371 ***	0.010	—	
7. RMET	0.022	0.051	0.009	−0.042	−0.023	0.040	—

Note. ** *p* < 0.01, *** *p* < 0.001. DERS: Difficulties in Emotion Regulation Scale—Short Form, ICS: Interpersonal Competence Scale, IRI: Interpersonal Reactivity Index, GSBP: Generic Scale of Being Phubbed, GSP: Generic Scale of Phubbing, RMET: Reading the Mind in the Eyes Test, SAS: Smartphone Addiction Scale Short Form.

**Table 3:** Comparison of fit indices for the full- and partial-mediation models.

Model	χ^2^	df	*p*	CFI	TLI	RMSEA	SRMR	AIC	BIC
Full-mediation	26.72	8	0.001	0.962	0.914	0.088	0.026	7548.36	7607.89
Partial-mediation	2.50	2	0.286	0.999	0.991	0.029	0.014	7536.14	7617.99

Note. CFI = Comparative Fit Index; TLI = Tucker–Lewis Index; RMSEA = Root Mean Square Error of Approximation; SRMR = Standardized Root Mean Square Residual; AIC = Akaike Information Criterion; BIC = Bayesian Information Criterion.

**Table 4:** Direct effects in the final structural path model.

Result Variable	Predictor	B	SE	β	*p*	%95 Bootstrap CI
SAS	DERS	0.275	0.038	0.353	**<** **0.001**	0.201, 0.350
(R^2^ = 0.313)	ICS	−2.975	1.227	−0.131	**0.015**	−5.434, −0.617
	DPUD	1.232	0.198	0.351	**<** **0.001**	0.859, 1.639
GSP	SAS	1.237	0.063	0.775	**<** **0.001**	1.113, 1.366
(R^2^ = 0.694)	DERS	−0.083	0.044	−0.066	0.064	−0.170, 0.002
	ICS	−5.270	1.488	−0.145	**<** **0.001**	−8.095, −2.369
	DPUD	0.458	0.185	0.082	**0.013**	0.100, 0.835
	IRI	0.072	0.059	0.041	0.228	−0.049, 0.183
	RMET	0.124	0.210	0.017	0.554	−0.301, 0.529
GSBP	SAS	0.198	0.142	0.100	0.162	−0.083, 0.468
(R^2^ = 0.088)	DERS	0.117	0.110	0.076	0.286	−0.095, 0.341
	ICS	1.470	2.790	0.032	0.598	−3.884, 6.983
	DPUD	0.521	0.420	0.074	0.215	−0.292, 1.346
	IRI	0.444	0.129	0.203	**0.001**	0.185, 0.690
	RMET	0.539	0.476	0.060	0.257	−0.376, 1.508

Note. DERS: Difficulties in Emotion Regulation Scale—Short Form, ICS: Interpersonal Competence Scale, IRI: Interpersonal Reactivity Index, GSBP: Generic Scale of Being Phubbed, GSP: Generic Scale of Phubbing, RMET: Reading the Mind in the Eyes Test, SAS: Smartphone Addiction Scale Short Form, DPUD: Daily Phone Usage Duration, B = unstandardized regression coefficient; SE = standard error; β = standardized regression coefficient; CI = confidence interval; R^2^ = proportion of variance explained. Bootstrap confidence intervals were calculated with 5000 resamples. Statistically significant coefficients are shown in bold.

**Table 5:** Indirect associations with bootstrap confidence intervals.

Indirect Effect	B	β	SE	%95 Bootstrap CI	*p*
DERS → SAS → GSP	0.341	0.273	0.053	0.239–0.449	**<** **0.001**
DERS → SAS → GSBP	0.055	0.035	0.040	−0.023–0.134	0.169
ICS → SAS → GSP	−3.680	−0.101	1.553	−6.868–−0.752	0.018
ICS → SAS → GSBP	−0.589	−0.013	0.522	−1.804–0.235	0.259
DPUD → SAS → GSP	1.523	0.272	0.244	1.063–2.017	**<** **0.001**
DPUD → SAS → GSBP	0.244	0.035	0.181	−0.102–0.618	0.178

Note. DERS: Difficulties in Emotion Regulation Scale—Short Form, ICS: Interpersonal Competence Scale, SAS: Smartphone Addiction Scale Short Form, GSBP: Generic Scale of Being Phubbed Scale, GSP: Generic Scale of Phubbing, DPUD: Daily Phone Usage Duration. Indirect effects were calculated using the 5000 bootstrap resampling method. B = unstandardized indirect effect coefficient; β = standardized indirect effect coefficient; SE = standard error; CI = confidence interval. The absence of zero in the 95% bootstrap confidence interval indicates that the indirect effect is statistically significant. Significant indirect effects are shown in bold.

**Table 6:** Total associations in the final structural path model.

Total Effect	B	β	SE	%95 Bootstrap CI	*p*
DERS → GSP	0.258	0.207	0.066	0.124–0.387	**<** **0.001**
DERS → GSBP	0.172	0.111	0.094	−0.014–0.360	0.068
ICS → GSP	−8.950	−0.246	1.984	−12.886–−5.039	**<** **0.001**
ICS → GSBP	0.880	0.019	2.854	−4.688–6.561	0.758
DPUD → GSP	1.981	0.353	0.318	1.378–2.631	**<** **0.001**
DPUD → GSBP	0.765	0.109	0.416	−0.056–1.596	0.066

Note. DERS: Difficulties in Emotion Regulation Scale—Short Form, ICS: Interpersonal Competence Scale, GSBP: Generic Scale of Being Phubbed, GSP: Generic Scale of Phubbing, DPUD: Daily Phone Usage Duration, Total effect refers to the sum of direct and indirect effects. B = unstandardized total effect coefficient; β = standardized total effect coefficient; SE = standard error; CI = confidence interval. Bootstrap confidence intervals. Statistically significant coefficients are shown in bold.

## Data Availability

The datasets used and analyzed during the current study are available from the corresponding author on reasonable request.

## Abbreviations

DERS	Difficulties in Emotion Regulation Scale—Short Form
ICS	Interpersonal Competence Scale
IRI	Interpersonal Reactivity Index
GSBP	Generic Scale of Being Phubbed
GSP	Generic Scale of Phubbing
RMET	Reading the Mind in the Eyes Test
DPUD	Daily Phone Usage Duration
